# Genome-Wide Association Study Identifies Candidate Genes That Affect Plant Height in Chinese Elite Maize (*Zea mays* L.) Inbred Lines

**DOI:** 10.1371/journal.pone.0029229

**Published:** 2011-12-28

**Authors:** Jianfeng Weng, Chuanxiao Xie, Zhuanfang Hao, Jianjun Wang, Changlin Liu, Mingshun Li, Degui Zhang, Li Bai, Shihuang Zhang, Xinhai Li

**Affiliations:** Institute of Crop Science, Chinese Academy of Agricultural Sciences, The National Key Facility for Crop Gene Resources and Genetic Improvement, Beijing, China; Michigan State University, United States of America

## Abstract

**Background:**

The harvest index for many crops can be improved through introduction of dwarf stature to increase lodging resistance, combined with early maturity. The inbred line Shen5003 has been widely used in maize breeding in China as a key donor line for the dwarf trait. Also, one major quantitative trait locus (QTL) controlling plant height has been identified in bin 5.05–5.06, across several maize bi-parental populations. With the progress of publicly available maize genome sequence, the objective of this work was to identify the candidate genes that affect plant height among Chinese maize inbred lines with genome wide association studies (GWAS).

**Methods and Findings:**

A total of 284 maize inbred lines were genotyped using over 55,000 evenly spaced SNPs, from which a set of 41,101 SNPs were filtered with stringent quality control for further data analysis. With the population structure controlled in a mixed linear model (MLM) implemented with the software TASSEL, we carried out a genome-wide association study (GWAS) for plant height. A total of 204 SNPs (*P*≤0.0001) and 105 genomic loci harboring coding regions were identified. Four loci containing genes associated with gibberellin (GA), auxin, and epigenetic pathways may be involved in natural variation that led to a dwarf phenotype in elite maize inbred lines. Among them, a favorable allele for dwarfing on chromosome 5 (SNP PZE-105115518) was also identified in six Shen5003 derivatives.

**Conclusions:**

The fact that a large number of previously identified dwarf genes are missing from our study highlights the discovery of the consistently significant association of the gene harboring the SNP PZE-105115518 with plant height (*P* = 8.91e-10) and its confirmation in the Shen5003 introgression lines. Results from this study suggest that, in the maize breeding schema in China, specific alleles were selected, that have played important roles in maize production.

## Introduction

Attaining high and stable yield has been one of the major goals in the production of crops, including maize. Semi-dwarfism, an important agronomic trait that contributes to crop yield, improves harvest index and nitrogen response, and increases lodging resistance [1]. For example, semi-dwarf genes, such as *sd1* in rice and *RHT* in wheat, led to the first Green Revolution in the 1960s [2]. Similarly, dwarf genes provided by the elite maize inbred line Shen5003 have been successfully used to develop other Chinese maize inbreds with reduced plant height [3]. One Shen5003 derivative, Zheng58, for example, is the parent of the maize hybrid ZhengDan958, which has been grown in an extensive area totaling ∼35 Mha in China.

Plant height is a complicated quantitative trait that is controlled by a large number of genes. Defects in either signaling or biosynthesis of GA or brassinosteroids (BR) may give rise to a typical dwarf phenotype. Semi-dwarfism in rice, controlled by *sd1*, results from a deficiency of *GA20-oxidase* activity in the GA biosynthetic pathway [4]. In wheat, semi-dwarfism can be controlled by the reducing-height (*RHT*) gene, which encodes a DELLA protein involved in the GA signaling pathway [2]. Recent genetic analysis and molecular characterization of dwarf mutants in maize revealed that mutations in dwarf genes including *d1*, *d2*, *d3*, *d5*, *d8*, *d9*, *An1*, *DWF1* and *DWF4* [5], [6], [7], [8], [9], [10], [11], [12], [13], lead to a dwarf phenotype. In addition, auxin synthesis and signalling, and epigenetic networks, also play important roles in stem elongation. For example, a multidrug resistant-like (MDR-like) ABC transporter involved in polar auxin transport leads to reduced plant height in the maize *br2* mutant [14]. An epigenetic effect is illustrated by *Epi-d1*, a spontaneous rice mutant that displays a metastable dwarf phenotype caused by silencing of the *DWARF1* (*D1*) gene, which encodes a GTP-binding protein involved in giberellin signalling [15]. However, due to the yield loss caused by various reproductive abnormalities associated with these genes or mutants, they have been difficult to use in breeding for dwarf stature in maize [16].

To date, using different mapping populations, more than 219 QTLs for plant height have been reported across ten maize chromosomes (2010 December update to Gramene database). One major locus consistently detected between bins 5.05 and 5.06 reduces height of maize inbreds from different bi-parental populations developed from Shen5003 and derived inbreds [17], [18], [19], and also has the same effect in the authors' previous study [20]. Among important loci, *ph3*, which is located on chromosome 5, accounts for 38.6% of the total phenotypic variation [17]. This locus should play an important role in future breeding for dwarfness in maize in China.

Several approaches such as map-based cloning and association mapping have been successfully used to identify candidate genes. *Teosinte branched1* (*Tb1*), which explains a large proportion of phenotypic variation in maize plant architecture, was first cloned by time-consuming positional cloning in maize [21]. Association mapping proceeds by assessing genetic and phenotypic variation in a population to establish newly discovered linkages between markers and particular phenotypes [22]. With the development of SNP assays and associated statistical methods, GWAS has also been used to scan for novel loci influencing human diseases [23], and is a useful adjunct to classical genetic mapping of quantitative traits in plants [24]. In *Arabidopsis*, Aranzana et al. were able to identify genes controlling natural variation in flowering time and pathogen resistance using genome-wide polymorphisms among 95 accessions [25]. Also, the power of GWAS for analyzing 107 phenotypes in *Arabidopsis* was demonstrated using 250,000 SNPs [26]. In rice, approximately 3.6 million SNPs were used to confirm six loci harboring previously identified genes for color, amylose content, and grain shape among sequences of 517 landraces [24]. Forty-eight genomic regions correlated to aluminum tolerance were identified by GWAS in a diverse collection of 383 rice accessions [27]. In barley, the *HvbHL H1* gene was fine-mapped by GWAS using a 1536-feature SNP array based on expressed sequence tags [28]. As an outcrossing species, abundant diversity and rapid linkage disequilibrium (LD) decay make maize an ideal crop for association mapping. Using a nested association mapping (NAM) panel, 1.6 million HapMap SNPs [29] have been used to test each SNP for effects on several traits, including flowering time [30], southern leaf blight [31], and leaf architecture [32]. Association analysis may also be used to identify traits controlled by selection-candidate genes [33]. Two types of maize SNP arrays have been constructed and commercialized for GWAS using populations of elite maize inbreds. One is the Illumina oligo pool assay that was developed from 582 candidate genes with 1536 SNPs, mainly for drought-related loci [34], [35]. Another is the MaizeSNP50 BeadChip with over 55,000 evenly spaced markers, covering two-thirds of predicted genes (19,350) in the maize genome with 1–17 SNPs/gene (Illumina, Inc.). Because LD decays more rapidly in outcrossing species than in selfing species, more markers were needed to scan the whole genome for associated loci in maize: 50,000 markers were required for elite maize lines, but 750,000 markers were required for diverse maize landraces [22].

So far, two kinds of genome-wide association panels have been constructed for association mapping studies in maize. The NAM panel, which comprises 5000 recombinant inbred lines derived from crossing the B73 reference line to 25 diverse inbred lines, was constructed to understand the molecular basis of phenotypic variation with great power [36]. Another panel is comprised of elite maize inbred lines, including 632 inbred lines representing global maize diversity [34], panels representative of American and European diversity [37], and 375 lines of mainly Chinese germplasm (data unpublished). The population structure of 187 inbred lines commonly used in Chinese maize breeding programs provided the basis for association mapping in this study [38].

There is, however, little GWAS information for SNP markers associated with dwarf loci in panels of elite inbred Chinese maize lines and germplasm, particularly for the widely used inbred line Shen5003. Therefore, the objectives of this study were to: 1) carry out a GWAS for plant height including major dwarf loci using 41,101 well-selected SNPs covering the entire maize genome; 2) identify loci for plant height in Chinese elite maize inbred lines under three environmental conditions; and 3) explore the genetics of major alleles at dwarf loci on chromosome 5 in Shen5003 and six of its derivatives. Finally, approaches for identifying candidate genes using GWAS in maize, in comparison with selfing species, are discussed.

## Results

### SNP calling results

Using the MaizeSNP50 BeadChip, a total of 50,195 SNPs (91.1%) were successfully called with less than 20% missing data across the 288 inbreds. Of these 50,195 SNPs, 2784 were monomorphic in all lines. The source sequences of the SNP regions were used for basic local alignment search tool (BLAST) comparisons with the MaizeGDB (B73 RefGen_v1) to update coordinate information. A total of 231 of the 50,195 successful assays did not have a BLAST match below the threshold of 1*e* ^−4^ and 2945 markers harbored multiple loci on maize chromosomes. Thus, a subset of 44,235 SNPs (80%) with known physical position was generated in this study.

Seven inbred lines with a high heterozygosity rate (HR) (>10%) across 44,235 markers were discarded. The remaining 281 lines with an average heterozygosity of 0.5% were used for further analysis. For these samples, the reproducibility between replicates across all data points was about 99.9%, which was consistent with high-quality data for the maize B73 line (Illumina, Inc.). Subsequently, the error rate (ER) for the genotyping was reduced from 0.0873% to 0.0353% by repeating assays in four line and discarding SNPs with multiple loci (Table 1).

**Table 1:** Quality evaluation of the MaizeSNP50 in this study.

	Repeat	Total	Qi319		Huangzaosi		Ye478		Dan340	
			1	2	1	2	1	2	1	2
A	CR(%)	95.04	94.94	95.56	94.13	94.91	96.04	96.43	94.79	95.54
	HR(%)	2.99	2.33	2.08	2.46	2.03	1.97	1.91	2.47	2.22
	ER(%)	0.0873	0.06		0.103		0.093		0.093	
B	CR(%)	98.5	98.6	98.76	98.03	98.3	99.13	99.16	98.59	98.75
	HR(%)	0.5	0.18	0.15	0.14	0.07	0.12	0.14	0.13	0.1
	ER(%)	0.0353	0.005		0.023		0.095		0.018	

Note: CR, Call Rate; HR, Heterozygosity Rate; ER, Error Rate.

A: 55,126 SNPs in 288 samples; B: 44,235 SNPs in 281 samples.

### Linkage disequilibrium in association panels

All 44,235 of the SNPs that were mapped *in silico* to the maize physical map across ten chromosomes were used to determine the extent of LD in the association panel. As shown in Table 2, the LD estimate is about 27.7 kb across all chromosomes. A shorter LD (25.4 kb) estimate is observed on chromosome 6, but the longest (29.2 kb) was observed on chromosome 3.

**Table 2:** Average LD decay distance of the 10 chromosomes for *r^2^* greater than 0.1.

Chromosome	LD decay(kb)
1	26.1
2	28.0
3	29.2
4	28.5
5	28.7
6	25.4
7	27.9
8	27.5
9	27.6
10	28.7
Average	27.7

### Genetic feature identified for plant height

The mean heritability for plant height across three environmental conditions was estimated as 70.63%, suggesting that this trait would respond well to artificial phenotypic selection [39]. Table 3 shows that there was greater phenotypic variation but a less significant genotype-by-environment effect in environment II. Phenotypic correlations for plant height among the three locations were all significant (*P*<0.01).

**Table 3:** Plant height of association panel across three environments.

Site	Num	Mean	Range(cm)	CV	Skew	Kurt	Correlation coefficient	
							I	II
I	190	178.2	106.4–253.1	14.11	0.11	0.01		
II	186	172.7	91.7–297.0	16.49	0.4	1.67	0.8551**	
III	187	118.8	76.2–164.7	15.4	0.22	−0.37	0.7275**	0.6959**

Note:

**indicate significance at p = 0.01.

I–III are the environments. = Jilin 2008, II = Beijing 2008, III = Sanya 2008.

### SNP markers and genes associated with plant height

**Figure 1:**
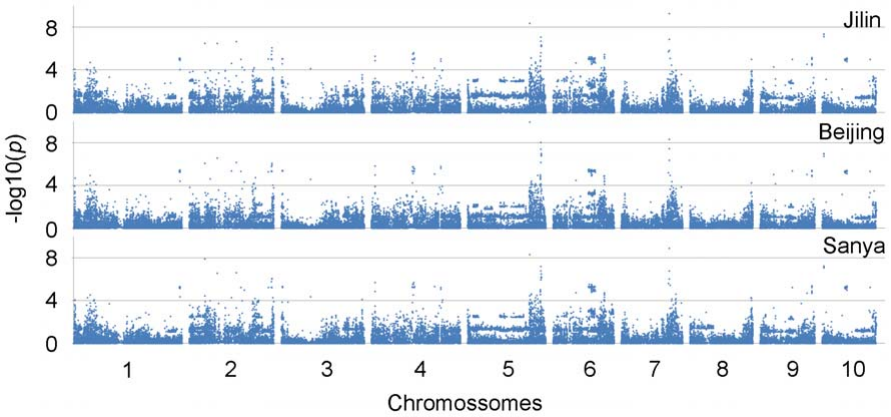
Genome-wide association study of plant height with mixed linear model. Four blocks (red boxes) harboring the candidate genes controlling plant height were identified across three environments (MAF≥0.05).

Approximately every fifth or sixth SNP was selected according to physical position from a collection of 5000 SNP markers and used to estimate population structure and relative kinship (Figure S1), and the power of these SNPs was similar to 500 SSRs [40]. Across three environments, taking the population structure into consideration, the mean value for −log10(*P*) was markedly decreased by about half (0.552551/0.973008) within a total of 41,101 SNPs, and the peaks for SNPs on chromosomes were reduced with MLM (Figure 1, S2). These data demonstrate that the excess of tenuous associations arising mostly from confounding effects of population structure were eliminated.

A total of 204 SNPs across 10 chromosomes were significantly associated with plant height (*P* ≤0.0001), among which SNP PZE-105115518 was the most significant (*P* = 8.91e-10). These 204 SNPs covered 105 genomic blocks harboring coding regions from which more than 225 cDNAs were identified. The data showed that 78.9% of the SNPs were surrounded by cDNAs and that eight SNPs were located in the fragments of genes predicted from the B73 reference sequence. Several hot spots, including bins 1.11, 2.09, 3.02, 4.05, 5.05, 5.06, 6.03, 6.04, 6.05, 9.07, and 10.03 were identified as containing more than three SNPs in the predicted genes (Table S1), probably due to the extended LD decay revealed by high-density markers. Notably, peak signals for GWAS loci closely linked to genes for a particular trait were significantly over-represented. Given our emphasis on the factors influencing plant height, four loci harboring candidate genes for plant height were located in bins 1.11, 5.05, 6.04, and 9.07. The gene (GenInfo Identifier, GI: 194692741) for SNP SYN21642 in bin 1.11 (−log10(*P*) = 5.26) was similar to *AUXIN RESISTANT* 1 in *Arabidopsis* [41]. The SNP PZE-105115518 in bin 5.05 (−log10(*P*) = 9.05) is located within a gene (GI: 100273912) annotated as a hypothetical protein, related to an *Arabidopsis* DNA glycosylase, loss of function of which causes a semi-dwarf phenotype [42]. SNP PZE-106064587 in bin 6.04 (−log10(*P*) = 5.38), is located upstream of a gene (GI: 194701035) similar to *Gibberellin 20 oxidase*. One homolog of the candidate gene (GI: 195636889), upstream of SNP PZE-109106743 (−log10(*P*) = 4.83), was annotated as a tetratricopeptide repeat (TPR) domain protein involved in cell division cycle in rice. Together, these prediction data showed that GA, auxin, and epigenetic pathways may all be involved in natural variation that led to a dwarf phenotype in elite maize inbred lines.

### Introgressed fragments from Shen5003 on chromosome 5

**Figure 2:**
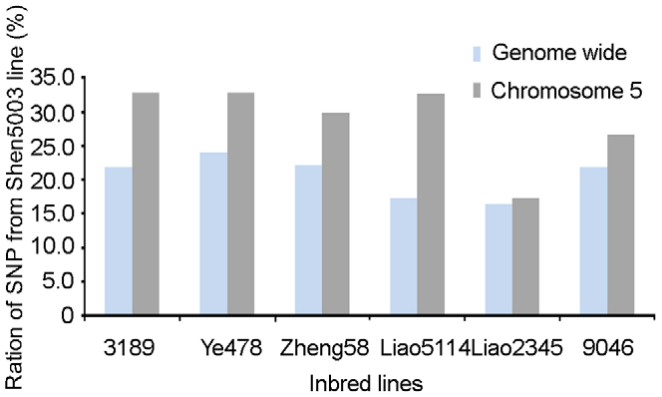
Distributions of Shen5003's alleles in 6 derivatives.

**Figure 3:**
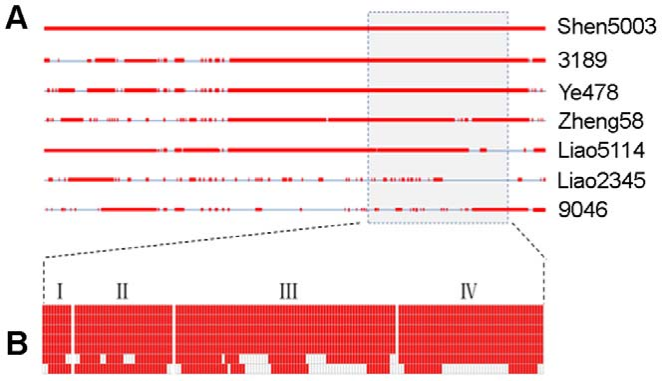
Distributions of introgressed fragments from Shen5003 in its derivatives on chromosome 5. (MAF≥0.05). A, chromosome 5; B, Four fragments at bin 5.05–5.06 harboring dwarf loci from Shen5003. Red bars indicate the fragments or SNPs from Shen5003, gap is chromatin from other lines.

Based on the pedigree of inbred lines, plant heights in six derivatives from Shen5003 were modified with this dwarf germplasm (Table 4). The introgressed fragments from Shen5003 could reduce plant height in these six derivatives from 10.5% to 25.77%, compared to the tall parent across all three environments tested (Table 4). Also, both the entire maize genome and chromosome 5 contain more alleles from Shen5003 than average in these derivatives (Figure 2). That is to say, several important traits, such as dwarfism, controlled by the introgressed fragments of this chromosome can be selected during the maize breeding process. Figure 3 shows that primarily four fragments between bins 5.05 and 5.06 from Shen5003 were transferred to the six derivatives. Most importantly, the introgressed fragment IV contains the SNP PZE-105115518, which is significantly associated with plant height (*P* = 8.91e-10) (Figure 1). No other genes with known dwarfing function were detected in these four fragments.

**Table 4:** Effect of introgressed fragments from Shen 5003, revealed by 6 derivatives across three environments.

Inbred lines	Source	Mean	I		II		III	
			Height(cm)	(%)	Height(cm)	(%)	Height(cm)	(%)
Shen5003		-	150.4	-	117.3	-	95.93	-
Tie7922		-	236	-	239.7	-	145.33	-
Liao2345	Tie7922×Shen5003	17.04	187.1	20.72	193.2	19.40	129.33	11.01
Liao5114	Tie7922×Shen5003	12.01	209.3	11.31	205.6	14.23	130.07	10.50
9046	Tie7922×Shen5003	18.39	201.5	14.62	182.3	23.95	121.2	16.60
8112		-	190.9	-	176	-	129.33	-
3189	U8112×Shen5003	21.68	155.3	18.65	137.7	21.76	97.47	24.63
Ye478	U8112×Shen5003	17.68	163.7	14.25	144.8	17.73	102.07	21.08
Zheng58	U8112×Shen5003×?	21.53	141.7	25.77	135	23.30	109.27	15.51

## Discussion

### Genome wide association study

Maize is an ideal candidate system for the application of GWAS with panels of inbred lines, because it has rapid LD decay as an outcrossing species and a high-quality reference genome sequence for generating enough informative markers [43]. In rice, the *GW2* gene, controlling grain width (GW) and weight, was undetectable via significant associations in the panel of 517 rice landraces tested [24]. However, two homologs of this gene, *ZmGW2-CHR4* and *ZmGW2-CHR5*, were significantly associated with kernel width (KW), or one of three other yield-related traits (kernel length (KL), kernel thickness (KT), and one-hundred kernel weight (HKW)) [44]. Previous studies have suggested that most SNPs associated with phenotype would be located very close to the causative genetic variant [45]. In our association panel, a total of 204 SNPs (*P*≤0.0001) and 105 genomic regions related to plant height were pinpointed with 41,101 SNPs (minor allele frequency, MAF≥0.05) across three environments. According to previous studies, only four genomic blocks harbored the known candidate genes controlling plant height (Figure 1, Table S1), the heritability of which is 70.63% in this panel. In rice, association signals for six traits, controlled by major loci were located close to known genes that were previously identified [24]. Similar findings were also identified in *Arabidopsis thaliana* [25], [26]. However, association signals for loci controlling heading date (flowering time) were not revealed on a whole-genome scale [24], [30]. These data suggest that the heritability of traits and locus-specific effects contributing to phenotypic variance should be the key factors in GWAS with maize inbred panels. In addition, for complex traits such as flowering time, a nested association mapping population has been constructed to identify evidence of numerous minor single-locus effects [30].

### Genetic architecture of plant height

Based on extensive results from dwarf mutants, GA and BR are major factors determining plant height [46]. Dwarfing effects of mutants in the GA biosynthesis and signaling pathways have been important for allowing manipulation of plant height during the first Green Revolution [2]. For example, *D1*, the gibberellin-responding dwarf gene of maize, controls the three biosynthetic steps: from GA20 to GA1, from GA20 to GA5, and from GA5 to GA3 [7]. The GA-insensitive dwarf genes *D8* in maize and *RHT* in wheat are both DELLA proteins with GTP-binding roles in GA signalling [2]. In our study, a total of 204 SNPs covering 105 genomic regions were significantly associated with plant height (*P*≤0.0001), and four known loci harboring candidate genes for plant height were located in bins 1.11, 5.05, 6.04, and 9.07, based on previous studies. However, only one SNP, PZE-106064587 in bin 6.04, was identified as closely linked to a predicted gene for a protein similar to gibberellin 20 oxidase. In addition, auxin signalling and epigenetic networks also play important roles in internode elongation. In the maize *br2* mutant, height reduction resulted from the loss of function of a P-glycoprotein that modulates polar auxin transport in the maize stalk [14]. Our results show a significant association between plant height and SNP SYN21642 in bin 1.11 across environments, located close to a gene similar to *AUXIN RESISTANT* 1 (*AXR*1). In the case of *Arabidopsis axr*1, the decrease in plant height is the consequence of a defect in auxin action [41]. Also, joint mapping suggested that there is a stable QTL for plant height between bin 1.10 and bin 1.11 [20], [47], [48], [49]. Moreover, SNP PZE-105115518 is located within a gene annotated as hypothetical protein related to the *Arabidopsis* DNA glycosylase, loss of function of which results a semi-dwarf phenotype [42]. This suggests that this gene in bin 5.05 may also participate in control of plant stature through an epigenetic pathway and would be a good candidate for manipulation of plant height in maize. These data indicate that the genetic network for dwarfness is composed of multiple pathways and that these SNPs reflect the genetic variation existing in our association panel. The new loci identified here are favorable candidates for subsequent studies to further our understanding of the genetic control of plant architecture including height in maize.

### Dwarf loci as revealed by the association panel

Breeding for semi-dwarfness was a key objective for high yield in the first Green Revolution [2], when it was discovered in maize, yield potential may be increased by reducing plant height and selecting for erect leaves [16]. However, most maize dwarf mutants are short, compact plants with shortened internodes, short wide leaves, and short erect tassels [50]. In this study, the effects of above-mentioned dwarf loci, such as *d1*, *d2*, *d3*, *d5*, *d8*, *d9*, *An1*, *DWF1*, and *DWF4* [5], [6], [7], [8], [9], [10], [11], [12], [13] were not detectable across our association panel. Furthermore, previous studies had indicated difficulties with anther extrusion or low yield for mutants in such key genes controlling plant height [16]. It had been suggested that mutagenesis of the maize gene *D8* would affect quantitative variation in maize plant height [2], however, no significant association between *D8* polymorphisms and plant height were found in the DELLA region, although this region was completely conserved across the tested lines [51]. Indeed, some mutations in key genes for plant height often cause extreme phenotypes in addition to reducing plant height and would be difficult to select by maize breeding and to exploit commercially.

**Figure 4:**
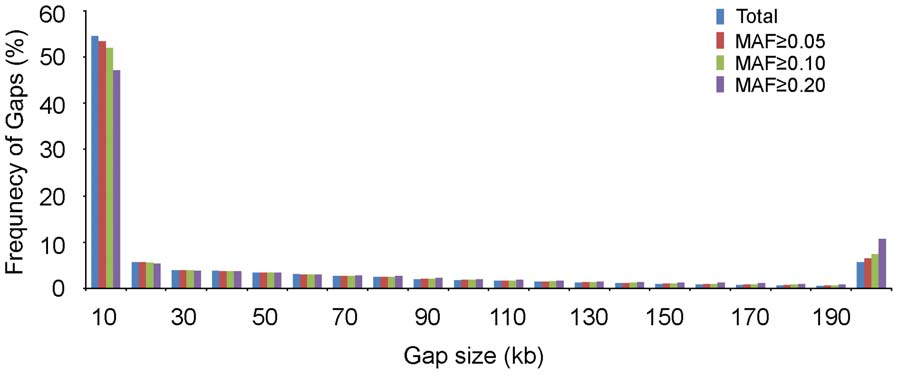
Probe spacing across the entire maize genome with different minor allelic frequencies.

To date, more than 219 quantitative trait loci for plant height have been detected (2010 December update to Gramene database), and a total of 204 SNPs were significantly associated with plant height (*P*≤0.0001) in this study. However, there are cases in which QTLs discovered in bi-parental mapping populations are not detected by GWAS. One reason for this is that the moderate number of founders has limited resolution to dissect low-frequency or subpopulation-specific alleles. For instance in rice, the peak signal for grain width was tied closely to a previously identified domestication gene, *GW5* [52], whereas no significant association was found for the sub-population-specific *GW2* gene [53] due to the lack of a wide range of alleles in theassociation panel [24]. Interestingly, two maize homologs of the *GW2* gene, *ZmGW2-CHR4*, and *ZmGW2-CHR5*, have been significantly associated with kernel-related traits [44]. Meanwhile, the GWAS that used 383 diverse rice accessions discovered 48 regions associated with Al tolerance, most of which were sub-population specific [27]. Other reasons for lack of detection of some alleles may be that LD may diminish rapidly with distance in some chromosomal regions, or that a set of 41,101 SNPs is unlikely to capture all of the haplotypes present in diverse maize inbred lines. Figure 4 indicates that more than 50% of the gaps between two adjacent SNPs are less than 10 kb, and the average marker density is one marker every 50 kb (Table S2). In commercial inbred lines, LD decay may be slower and distance between markers may extend to more than 100 kb [54], while the average LD decay in our association panel is about 27.7 kb across ten chromosomes. Also, previous studies showed that the LD decay distance ranged from 5 to 10 kb among chromosomes in a set of global maize inbred lines [34], to 250 bp within the *PSY2* gene in maize [55], which indicated that a higher density of markers should be used to adequately cover the whole genome in GWAS for certain populations. Finally, incorrect prediction, or imputation, of unobserved genotypes among SNPs in an association panel could reduce the accuracy of association estimates [32]. With the panel used in this study, there was a 0.0873% error rate for 55,126 SNP markers, while there was 0.0353% error rate in the final dataset (Table 1). That is to say, SNP accuracy was high for association mapping in this study. Despite these limitations, four candidate genes prevalent in maize breeding have been identified and validations by joint mapping are in progress.

To summarize, GWAS identified more phenotype–genotype associations and provided higher resolution with high-density SNP markers, but QTL mapping identified sub-population-specific alleles not detected by GWAS.

### Dwarf maize germplasm in China

Compared with other maize cropping system around the world, the summer maize zone in China, which accounts for 35.6% of the national maize-growing areas and 35% of national total output [56], is unusual for its annual double-cropping rotation with wheat. Usually, the number of growing days for varieties in this zone is less than 110 days. Therefore, the traits associated with plant height, such as short vegetative growth, high harvest index, early maturity, and lodging resistance may explain the use of varieties derived from one key dwarf donor line that has always existed in this specific growing region. The favorable dwarf allele harbored by Shen5003, which was derived from the American hybrid cultivar 3174, has been widely utilized in China, particularly in the Yellow and Huai River summer maize zones. And Ye478, the variety with a genetic background most similar to that of Shen5003 (Figure 2), has been used to develop more than 30 inbred lines and about 58 hybrids in China. Zheng58, one of these derived lines, is one of the parents of the commercial hybrid ZhengDan958, the most widely grown cultivar in China. The major QTL allele from Shen5003 between bins 5.05 and 5.06 that reduces plant height has been consistently detected across all three environments [20] in the Ye478×Dan340 F_2∶3_ population, in addition to other bi-parental populations [17], [18], [19]. SNP PZE-105115518, identified in bin 5.05 in the introgressed fragment from Shen5003, bears significant association with plant height. Altogether, our work shows that a favorable allele for the dwarf trait on chromosome 5 of great agronomic importance to maize breeding and production may have been previously selected from hybrid 3174 through Shen5003 during maize breeding in China.

## Materials and Methods

### Ethics Statement

All research was conducted using existing databases and permission for use of farm was granted when necessary by the Ministry of Agriculture of the People's Republic of China. In 2008, a subset of 190 of the 284 diverse inbred lines used for the association panel was grown across three locations, I, Gongzhuling, Jilin Province (43°30′N, 124°48′E), II, Shunyi, Beijing (40°07′N, 116°39′E) and III, Sanya, Hainan Province (18°45′N, 109°30′E). These locations were under the control of the Ministry of Agriculture of the People's Republic of China, and the required permission was obtained before these sites were used for planting.

### Plant materials and phenotyping of plant height

For genotyping and SNP-calling, a total of 284 diverse inbred lines (including the lines published in our previous report [38] with partial results unpublished) were used, which represented six subpopulations identified by population structure analysis: BSSS (American BSSS including Reid), PA (group A germplasm derived from modern U.S. hybrids in China), PB (group B germplasm derived from modern US hybrids in China), Lan (Lancaster Surecrop), LRC (derivative lines from Lvda Red Cob, a Chinese landrace), and SPT (derivative lines from Si-ping-tou, a Chinese landrace). Four lines (Qi319, Huangzaosi, Ye478, and D340), as quality controls, were used in six independent BeadChip panels.

A subset of 190 of the 284 diverse inbred lines in the panel was grown across three locations. A randomized complete-block design with three replications was employed, in which each line was planted in a plot of 20 plants in a 4.5-m long row with 0.6-m spacing between rows. Normal agronomic practices for maize were used in field management. At maturity, data for plant height from the soil surface to the base of anthers was collected, and the mean value for ten plants from each line was used for further statistical analysis.

Broad-sense heritability (*h^2^*) for plant height across three environments was computed according to Knapp (1985) [57]. The heritability was calculated as follows: *h^2^* = *σg^2^*/(*σg^2^*+*σgl^2^/n*+*σe^2^/nr*), where *σg^2^* is the genetic variance, *σgl^2^* is genotype-by-environment interaction, *σe^2^* is the error variance, *r* is the number of replications, and *n* is the number of locations. The estimates for *σg^2^*, *σgy^2^*, and *σe^2^* were obtained by analysis of variance (ANOVA) using the general linear model procedure of the statistical software SPSS 12.0 (IBM SPSS Inc.). Correlation coefficients were obtained with the program PROC CORR of SAS 8.0 (SAS Inc., Cary, NC).

### Genotyping

SNP genotyping was performed using the MaizeSNP50 BeadChip produced by Emei Tongde (Beijing). The SNP content featured on this chip, using 56,110 evenly spaced markers to cover the whole maize genome based on the B73 reference sequence, was selected from several public and private sources with 984 negative controls. These markers have been successfully validated in more than 30 diverse lines. DNA from 284 lines was extracted by a modified CTAB procedure according to Murray and Thompson (1980) [58], and the DNA quality for each sample was checked carefully before genotyping by gel-electrophoresis and spectrophotometer (Nanodrop 2000, Thermo Scientific). The DNA from each of four controls was divided into two samples, respectively. The SNP genotyping of 288 samples in six independent BeadChip panels was analyzed by the Infinium® HD assay ultra-protocol guide (Illumina, Inc.).

### SNP filtering

The selected 44,235 SNPs showed a wide distribution in minor allele frequencies, ranging from nearly monomorphic (MAF<0.5%) to equal allele frequency (MAF≈50%) across the 277 inbred lines (Figure S3). About 7.08% of SNPs have MAFs below 5%, with a roughly equal chance of being on any chromosome (Figure S4). As SNPs with low MAF usually produced unstable results in our preliminary data analysis, these markers (MAF<5%) were excluded from the association analysis, leaving 41,101 SNPs. Within this set of high-quality SNPs, the average marker density was one marker every 50 kb and 53.48% of SNPs were within a 10 kb interval of the neighboring markers (Figure 4).

### Genotype-phenotype association mapping

The linkage disequilibrium measurement parameter *r^2^* (*r* ^2^≥0.1) was used to estimate LD between all SNPs with less than 20% missing data on each chromosome via the software PLINK [59]. The alleles for all SNPs were calculated using PowerMarker 3.25 [60]. The population structure and kinship information for 277 lines were estimated with a mixed linear model using the software STRUCTURE version 2.3 [61] and SPAGeDi [62] with 5000 SNPs (MAF≥0.2), respectively. STRUCTURE was run to test K = 6 according to our previous study [38] three times with a burn-in period of 500,000 and 500,000 replications. The general linear model (GLM) and mixed linear model implemented in the program TASSEL version 3.0 [63] were used for genome-wide association mapping with 41,101 SNPs (MAF≥0.05), from which SNPs with –log10(*P*)≥4 were selected for candidate gene analysis.

### Candidate gene analysis

The sequence available for the maize line B73 provides a reference genome that can be used to analyze candidate genes [43]. BLAST against MaizeGDB was performed with 120 bp source sequence for SNP probes (Illumina, Inc.). The 30 kb window (the average LD decay is about 27.7 kb) was selected to fall within the estimated window of LD decay in our association panel. The genes within this window size were identified through MaizeGDB according to the positions of the closest flanking SNPs (*P*≤0.0001) or supporting intervals (http://gbrowse.maizegdb.org/cgi-bin/gbrowse/maize_v1/). The function of cDNAs were predicted using the blastx program at the National Center for Biotechnology Information (NCBI) database (http://blast.ncbi.nlm.nih.gov/).

## Supporting Information

Figure S1 **Population structure across 277 lines with 5000 SNPs (MAF≥0.2).**
(TIF)

Figure S2 **Genome-wide association studies on plant height with general linear model across three environments (MAF≥0.05).**
(TIF)

Figure S3 **Minor allelic frequency for 44,235 SNPs in maizeSNP50.**
(TIF)

Figure S4 **SNP distribution across the maize genome (MAF≥0.05).**
(TIF)

Table S1 **The SNPs, harboring cDNA, were detected with MLM in each environment (−log10(** ***P*** **)≥4, MAF≥0.05).**
(DOC)

Table S2 **SNP distribution of the Illumina maizeSNP50 within 277 inbred lines.**
(DOC)
