# Supplementary figures and images for: Genome-Wide Association Study Identifies Candidate Genes That Affect Plant Height in Chinese Elite Maize (Zea mays L.) Inbred Lines
Source: PLoS One. 2011 Dec 28;6(12):e29229. doi: 10.1371/journal.pone.0029229 (PMC3247246; doi:10.1371/journal.pone.0029229)

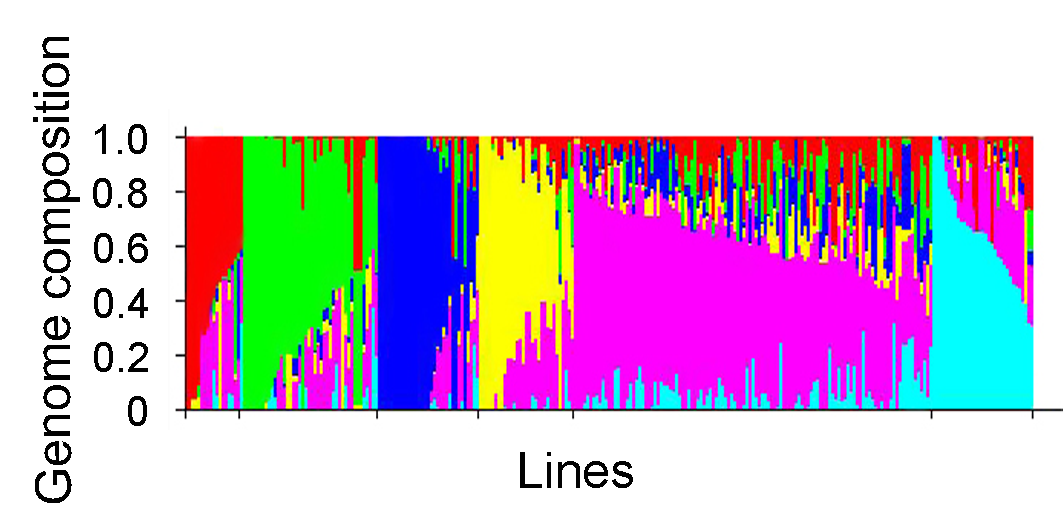

Supplement: Figure S1 — Population structure across 277 lines with 5000 SNPs (MAF≥0.2). (TIF) [file pone.0029229.s001.tif]

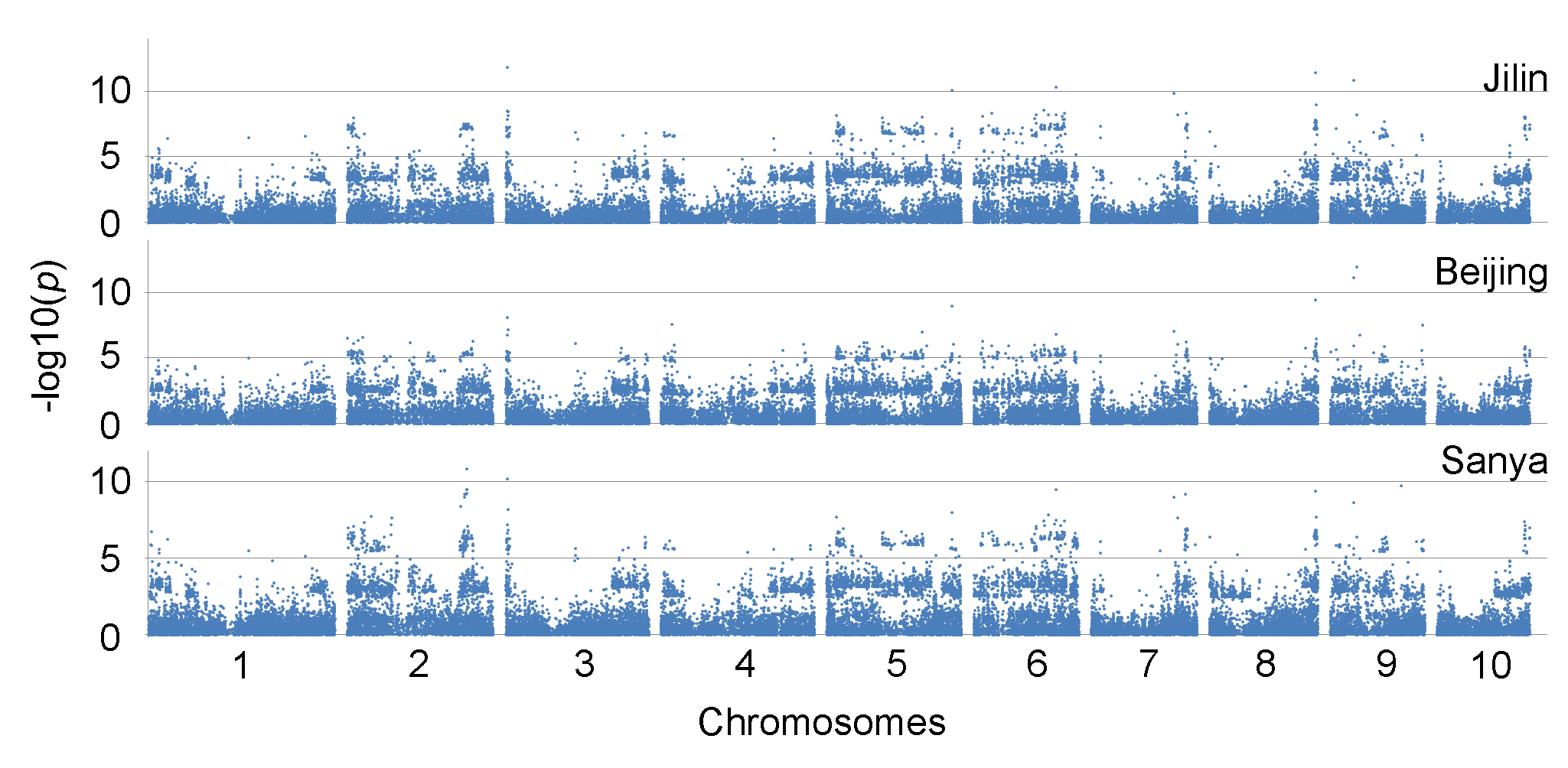

Supplement: Figure S2 — Genome-wide association studies on plant height with general linear model across three environments (MAF≥0.05). (TIF) [file pone.0029229.s002.tif]

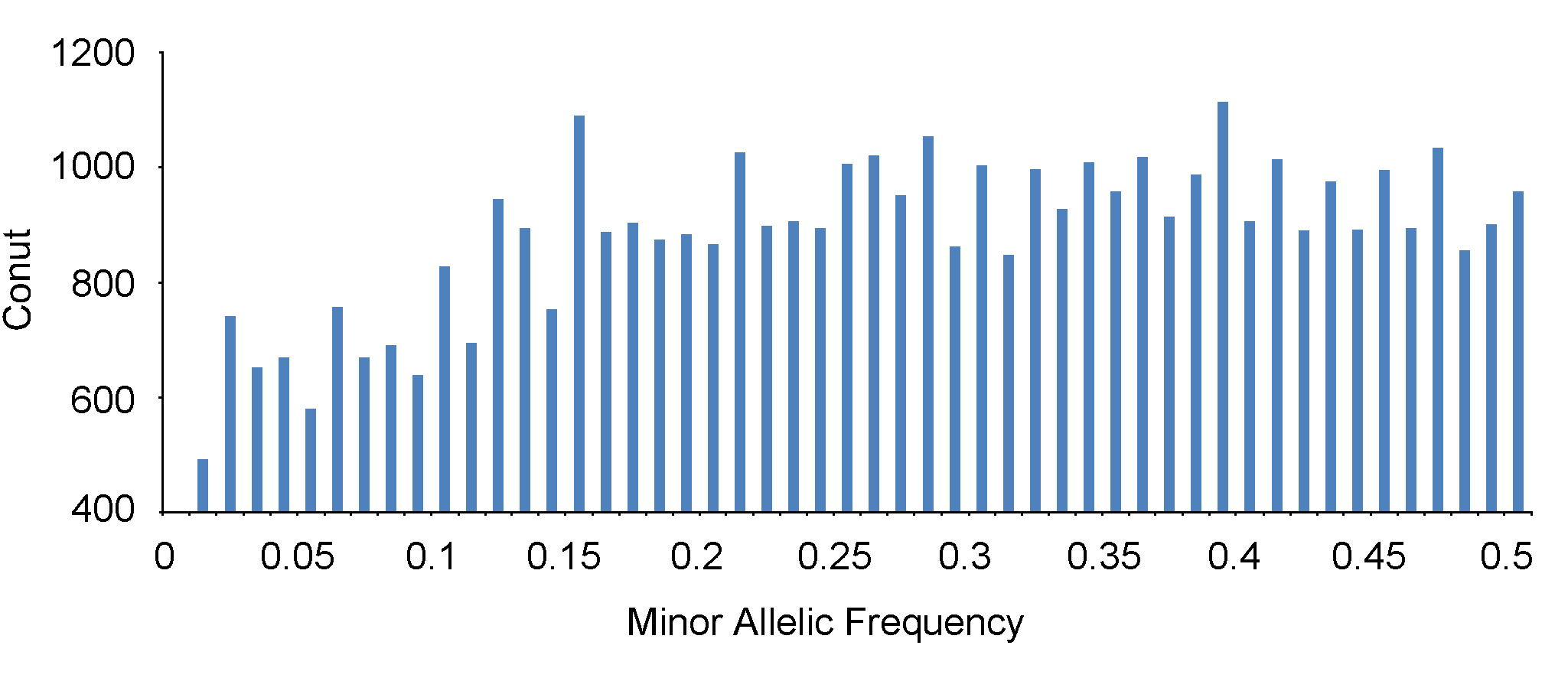

Supplement: Figure S3 — Minor allelic frequency for 44,235 SNPs in maizeSNP50. (TIF) [file pone.0029229.s003.tif]

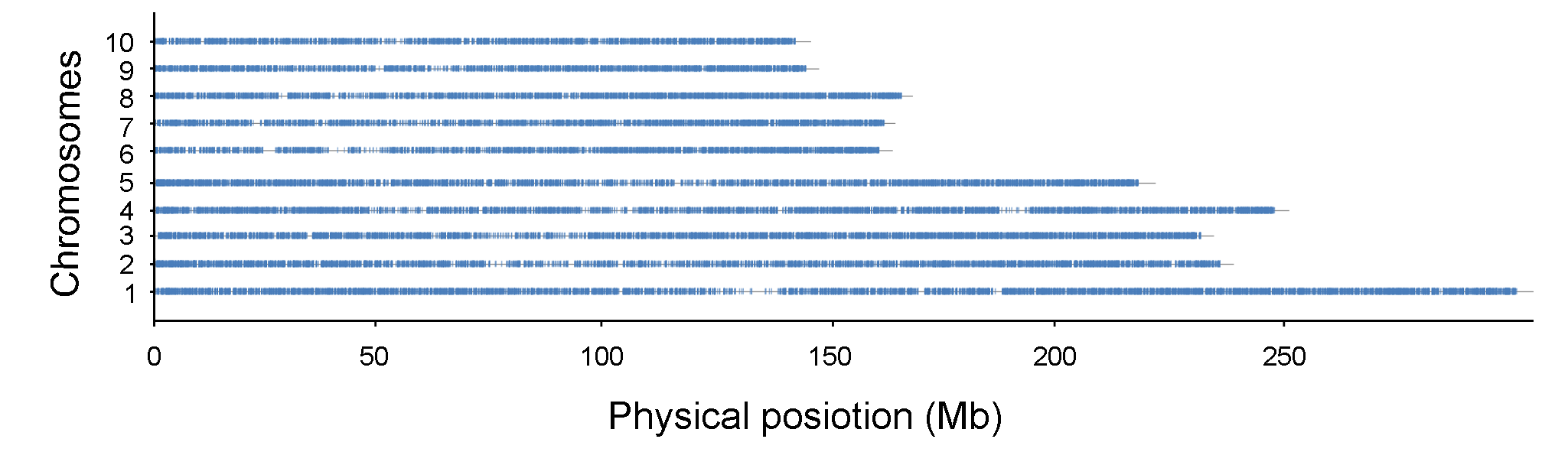

Supplement: Figure S4 — SNP distribution across the maize genome (MAF≥0.05). (TIF) [file pone.0029229.s004.tif]
