# Supplementary material for: Genome-Wide Association Study Identifies Candidate Genes That Affect Plant Height in Chinese Elite Maize (Zea mays L.) Inbred Lines
Source: PLoS One. 2011 Dec 28;6(12):e29229. doi: 10.1371/journal.pone.0029229 (PMC3247246; doi:10.1371/journal.pone.0029229)
Supplement: Table S1 — The SNPs, harboring cDNA, were detected with MLM in each environment (−log10( P )≥4, MAF≥0.05). (DOC) [file pone.0029229.s005.doc]

**Table S1.** The SNPs, harboring cDNA , were detected with MLM in each environment (-log10(*P*)≥4, MAF≥0.05)

| **Chr.Bin.** | **SNP context** | **MAF** | **Position** | **-log10（*p*）** | **Genebank ID of cDNA** |
| --- | --- | --- | --- | --- | --- |
| 1.03 | SYN23751 | 0.07 | 45,667,205 | 4.77 | 224032602;21207683 |
| 1.11 | PZE-101249180 | 0.41 | 293,033,956 | 5.23 | 195623013;195635234;194699209;194692741 |
|  | SYN21642 | 0.24 | 293,148,015 | 5.26 |  |
|  | SYN21645 | 0.23 | 293,149,275 | 5.26 |  |
| 1.11 | PZE-101249551 | 0.3 | 293,572,666 | 5.26 | 194703531;219884796;194701401;195598615;195623629; 195643563; |
|  | SYN31870 | 0.16 | 293,631,724 | 5.34 | 195650684;77817084;194697821;195599351; 195651654;195614029; |
|  | PUT-163a-60343270-2487 | 0.07 | 293,632,378 | 5.26 | 238011813 |
|  | PUT-163a-60343270-2483 | 0.43 | 293,632,416 | 5.30 |  |
|  | PZA00623.3 | 0.07 | 293,632,633 | 5.26 |  |
|  | PZE-101249607 | 0.07 | 293,643,189 | 5.26 |  |
|  | PZE-101249681 | 0.47 | 293,789,298 | 5.24 |  |
|  | PZE-101249703 | 0.47 | 293,797,834 | 5.26 |  |
|  | SYN26282 | 0.26 | 293,827,868 | 5.20 |  |
|  | SYN26278 | 0.23 | 293,907,712 | 5.26 |  |
|  | PZA02359.10 | 0.36 | 293,907,804 | 5.26 |  |
|  | SYN26276 | 0.2 | 293,909,016 | 5.27 |  |
|  | SYN16869 | 0.36 | 293,965,881 | 5.30 |  |
|  | SYN16863 | 0.35 | 293,966,055 | 5.29 |  |
|  | SYN16865 | 0.47 | 293,970,712 | 5.31 |  |
|  | SYN16866 | 0.21 | 293,970,802 | 5.30 |  |
|  | SYN16870 | 0.41 | 293,970,884 | 5.27 |  |
|  | SYN16864 | 0.26 | 293,971,360 | 5.26 |  |
|  | SYN16858 | 0.31 | 293,972,310 | 5.20 |  |
|  | SYN16859 | 0.3 | 293,972,376 | 5.26 |  |
|  | SYN16857 | 0.32 | 293,975,444 | 5.26 |  |
| 1.11 | SYN16861 | 0.48 | 294,012,926 | 5.23 | None |
| 1.11 | SYN8232 | 0.36 | 294,016,785 | 5.34 | 194689629;224031194;195638955 |
| 1.11 | SYN8230 | 0.2 | 294,225,180 | 5.29 | None |
|  | PZE-101250478 | 0.35 | 294,284,308 | 4.34 |  |
|  | PUT-163a-76012177-3730 | 0.32 | 294,310,784 | 5.29 |  |
| 1.11 | SYN410 | 0.21 | 294,334,287 | 5.28 | 224035770 |
|  | SYN408 | 0.26 | 294,334,673 | 5.27 |  |
| 2.04 | PZE-102064901 | 0.25 | 43,387,545 | 6.92 | 195651760;195622209;21211420;77816971;195635284; 195649548; |
|  |  |  |  |  | 224031322;195631106;195650634;195598113; 194691629;223942510; |
|  |  |  |  |  | 195616617 |
| 2.05 | PZE-102086050 | 0.44 | 77,722,833 | 6.61 | 21210229;195618491 |
| 2.05 | PZE-102100766 | 0.08 | 117,621,745 | 4.82 | None |
| 2.05 | PZE-102106143 | 0.08 | 131,091,915 | 6.51 | None |
| 2.05 | SYN32629 | 0.37 | 143,255,243 | 5.26 | None |
| 2.06 | PZE-102116835 | 0.08 | 152,716,127 | 4.51 | None |
| 2.09 | PUT-163a-14245519-344 | 0.49 | 219,833,963 | 5.27 | None |
| 2.09 | PZE-102186718 | 0.36 | 227,861,563 | 5.25 | 195603632;77817352;195610381 |
|  | PZE-102186765 | 0.46 | 227,871,357 | 5.30 |  |
| 2.09 | PZA02727.1 | 0.34 | 227,921,381 | 5.26 | 77817352;195594205;223929923 |
| 2.09 | PZE-102187403 | 0.26 | 228,325,622 | 5.75 | 219885916 |
| 2.09 | SYN37220 | 0.45 | 229,379,024 | 6.11 | 194696721;195600498 |
|  | SYN37212 | 0.45 | 229,380,553 | 5.99 |  |
|  | SYN37214 | 0.13 | 229,447,361 | 5.27 |  |
| 2.09 | PZE-102191957 | 0.28 | 231,389,415 | 4.19 | None |
| 3.01 | PZE-103006490 | 0.13 | 3,646,499 | 5.32 | 195618693;223942548;77812772;195605755;189127516 |
| 3.02 | SYNGENTA14279 | 0.14 | 4,327,117 | 5.31 | 219887540;195654336;54651627;195624679;224032882; |
|  | PUT-163a-71423695-3220 | 0.3 | 4,391,526 | 5.31 | 195639063;195625003;195654212;238008129 |
|  | PUT-163a-71423695-3217 | 0.3 | 4,391,624 | 5.31 |  |
|  | PUT-163a-71423695-3221 | 0.3 | 4,391,692 | 5.31 |  |
| 3.04 | PZE-103059322 | 0.46 | 81,792,618 | 4.43 | None |
| 4.02 | PZE-104011070 | 0.45 | 9,655,739 | 4.99 | 195653200；21206904 |
| 4.02 | PZE-104011716 | 0.47 | 10,155,668 | 5.68 | None |
| 4.05 | PZE-104059124 | 0.11 | 113,488,676 | 5.30 | 195613671;195622779;194696501;195636971;195651886 |
|  | PZE-104059186 | 0.11 | 113,679,507 | 5.30 |  |
|  | PZE-104059215 | 0.11 | 113,789,485 | 5.30 |  |
|  | PZE-104059275 | 0.1 | 113,973,538 | 5.26 |  |
|  | PZE-104059370 | 0.12 | 114,242,564 | 5.54 |  |
| 4.05 | PZE-104059554 | 0.12 | 114,810,604 | 5.54 | None |
| 4.05 | PZE-104059730 | 0.12 | 115,280,352 | 5.54 | 149014238 |
| 4.05 | PZE-104059995 | 0.34 | 116,071,954 | 4.82 | 195626799 |
| 4.05 | PZE-104060081 | 0.12 | 116,379,998 | 5.54 | 21209780 |
| 4.05 | PZE-104060254 | 0.14 | 116,878,111 | 5.58 | None |
| 4.05 | PZE-104060760 | 0.1 | 118,281,402 | 5.26 | 194700209 |
|  | PZE-104060808 | 0.06 | 118,489,740 | 5.28 |  |
|  | PUT-163a-101393073-23 | 0.21 | 118,508,702 | 5.26 |  |
| 4.05 | PZE-104061623 | 0.37 | 120,260,939 | 5.42 | None |
| 4.07 | PZE-104099262 | 0.05 | 174,834,024 | 4.93 | None |
| 4.08 | PZE-104111953 | 0.42 | 192,048,048 | 5.44 | 195650038;238008401;223974596;9716501;194694185; |
|  | PZE-104112105 | 0.39 | 192,625,346 | 5.26 | 224028372;21213104;195593277;238014447;224033270;77818602 |
| 5.05 | PZE-105115518 | 0.1 | 171,587,653 | 9.05 | 100273912 |
| 5.05 | PZE-105119892 | 0.08 | 175,753,812 | 4.66 | None |
| 5.05 | SYN32462 | 0.09 | 176,474,777 | 4.34 | 194702399;195626965;224030568;195616761;195628633;195621695 |
| 5.05 | SYNGENTA6857 | 0.19 | 187,806,163 | 4.42 | 195651516 |
| 5.05 | SYN16496 | 0.19 | 187,813,883 | 4.26 | None |
| 5.06 | PZE-105140135 | 0.09 | 194,054,746 | 4.51 | 195627543;195641071;195624163 |
| 5.06 | SYN35167 | 0.2 | 201,207,792 | 4.63 | 219886888;195649152;195600895 |
|  | SYN35180 | 0.2 | 201,222,026 | 4.94 |  |
|  | SYN35179 | 0.2 | 201,222,043 | 4.63 |  |
| 5.06 | SYN35181 | 0.31 | 201,231,896 | 4.94 | None |
| 5.06 | SYN14918 | 0.17 | 202,177,642 | 4.89 | 224028870;195642771;77818799 |
|  | SYN22059 | 0.14 | 202,348,251 | 5.89 |  |
| 5.06 | SYN21716 | 0.15 | 203,231,636 | 7.52 | 195611391;195651622;223945654;195618945;223946350; |
|  | SYN21722 | 0.08 | 203,237,282 | 7.06 | 195601975;21206831 |
| 5.06 | PZE-105154136 | 0.16 | 203,526,438 | 6.45 | None |
| 5.06 | PZE-105154147 | 0.16 | 203,552,121 | 6.50 | 21213138;224034522;195614415 |
| 5.06 | SYN1873 | 0.15 | 203,656,450 | 6.40 | 195599038;21212306;195607463;224029610;238015145; |
|  | SYN1883 | 0.17 | 203,657,409 | 6.65 | 195607463;224029610;224028836 |
|  | SYN1884 | 0.17 | 203,657,412 | 6.65 |  |
| 6.01 | PZE-106027217 | 0.21 | 64,071,503 | 4.70 | 194704095;195651538;195656346 |
| 6.03 | ZM012949-0478 | 0.37 | 98,110,091 | 5.35 | 54652576;195599533;219884530;194705703 |
| 6.03 | PZE-106049264 | 0.37 | 98,446,275 | 5.37 | 224029350;195643385;195645959;223947320;195652616 |
| 6.03 | PZE-106049550 | 0.41 | 98,812,016 | 5.29 | 195598132;89892726 |
|  | PZE-106049605 | 0.16 | 98,818,869 | 5.27 |  |
|  | PZE-106049631 | 0.22 | 98,922,186 | 5.29 |  |
|  | PZE-106049634 | 0.22 | 98,922,260 | 5.29 |  |
| 6.03 | PZE-106050102 | 0.3 | 100,145,296 | 5.52 | 195646691 |
|  | PZE-106050109 | 0.46 | 100,145,644 | 5.26 |  |
| 6.03 | SYN22604 | 0.46 | 102,553,600 | 5.30 | 195641921;21214762;194702729 |
| 6.03 | PZE-106052709 | 0.32 | 103,376,075 | 5.37 | 195655930;195606533;21213395;195647126;195654080; 2240329 |
|  | PZE-106052721 | 0.18 | 103,379,706 | 5.37 |  |
| 6.04 | SYN12087 | 0.19 | 104,662,488 | 5.28 | 223947740;194688287;195624721;157885766;194707037;195640367 |
|  | PZE-106053973 | 0.27 | 104,827,794 | 5.26 |  |
|  | SYNGENTA12137 | 0.28 | 104,913,649 | 5.26 |  |
| 6.04 | PZE-106054500 | 0.25 | 105,238,483 | 5.08 | 195615657 |
| 6.04 | SYN29719 | 0.36 | 105,772,731 | 5.27 | None |
| 6.04 | PZE-106055606 | 0.21 | 106,080,985 | 5.27 | 194693829 |
| 6.04 | PZE-106055949 | 0.21 | 106,474,307 | 4.86 | 195648331 |
|  | SYN28491 | 0.35 | 106,549,583 | 4.82 |  |
| 6.04 | SYN28493 | 0.39 | 106,561,457 | 5.49 | None |
|  | PUT-163a-71425802-3229 | 0.49 | 106,563,664 | 5.37 |  |
|  | PUT-163a-71425802-3226 | 0.48 | 106,563,676 | 5.30 |  |
| 6.04 | PZE-106056341 | 0.11 | 106,887,274 | 5.23 | 195628569;195612605;194707127 |
| 6.04 | SYN21153 | 0.39 | 107,251,764 | 4.92 | None |
|  | SYN21155 | 0.25 | 107,251,946 | 5.33 |  |
| 6.04 | PUT-163a-31909945-2005 | 0.5 | 107,666,209 | 5.36 | None |
|  | PUT-163a-31909945-2002 | 0.5 | 107,666,299 | 5.36 |  |
| 6.04 | PZE-106057097 | 0.16 | 107,978,164 | 5.40 | None |
|  | PZE-106057131 | 0.2 | 107,979,601 | 4.90 |  |
| 6.04 | PZE-106057176 | 0.28 | 107,989,037 | 5.25 | None |
|  | PUT-163a-18168669-1322 | 0.13 | 107,990,523 | 5.26 |  |
| 6.04 | PZE-106057592 | 0.16 | 108,425,358 | 5.25 | 223949984;194691679 |
| 6.04 | PZE-106057740 | 0.47 | 108,625,175 | 5.30 | None |
| 6.04 | PZE-106059969 | 0.23 | 110,897,582 | 5.18 | 21213664;194697037;2380085515 |
| 6.04 | SYN24768 | 0.2 | 111,001,556 | 5.12 | None |
| 6.04 | PZE-106060557 | 0.27 | 111,313,665 | 5.31 | 194693369;238012777;21209434 |
| 6.04 | PZE-106060834 | 0.48 | 111,815,960 | 5.07 | 195593152 |
| 6.04 | PZE-106061929 | 0.13 | 112,832,425 | 5.08 | 189468612;13447788 |
|  | SYN38139 | 0.32 | 112,959,088 | 5.26 |  |
| 6.04 | PZE-106062506 | 0.4 | 113,421,632 | 5.38 | None |
| 6.04 | PZE-106063088 | 0.05 | 114,587,494 | 5.32 | 195647449 |
|  | PZE-106063093 | 0.17 | 114,591,116 | 5.36 |  |
| 6.04 | SYN6717 | 0.19 | 115,138,621 | 5.13 | 195631104;188013427;21208698;223948098 |
| 6.04 | SYN6713 | 0.11 | 115,231,519 | 5.23 | None |
| 6.04 | PZE-106064587 | 0.3 | 116,494,814 | 5.38 | 194701035;219888188 |
| 6.04 | PZE-106064672 | 0.22 | 116,712,176 | 5.36 | 195602682;238008925;195655728 |
| 6.04 | PZE-106065285 | 0.3 | 117,711,437 | 5.01 | None |
| 6.05 | SYN24786 | 0.1 | 141,464,662 | 4.42 | 195611753;54654024;238011043;195614769 |
| 6.05 | PZE-106084780 | 0.29 | 142,271,782 | 4.63 | 238013049;238010135;195614473;195619487;194696999;195638585 |
|  | PZE-106084804 | 0.27 | 142,282,324 | 4.84 |  |
|  | SYN37576 | 0.23 | 142,284,853 | 4.06 |  |
|  | PZE-106084808 | 0.37 | 142,286,222 | 4.77 |  |
|  | SYN13667 | 0.25 | 142,397,587 | 4.16 |  |
|  | PZE-106084862 | 0.26 | 142,406,907 | 5.03 |  |
| 7.03 | PUT-163a-148967576-625 | 0.41 | 130,394,758 | 5.54 | None |
| 7.03 | PZE-107083353 | 0.16 | 132,457,905 | 8.88 | 194692043 |
|  | PZE-107083365 | 0.07 | 132,496,045 | 6.76 |  |
| 7.03 | PZE-107083402 | 0.3 | 132,580,248 | 6.52 | None |
| 7.03 | PZE-107085734 | 0.1 | 135,955,126 | 5.19 | None |
| 8.08 | PUT-163a-37416849-2022 | 0.08 | 170,971,822 | 4.49 | None |
| 9.03 | PUT-163a-4730575-2150 | 0.31 | 89,394,877 | 5.29 | None |
| 9.05 | PZE-109085277 | 0.19 | 129,574,572 | 4.82 | None |
| 9.07 | PZE-109106186 | 0.36 | 143,618,488 | 4.83 | 195653310;195597100;195641041 |
| 9.07 | PZE-109106215 | 0.26 | 143,627,910 | 5.33 | 195629811;223943754;195657810;223949274;195636889 |
|  | SYN32936 | 0.22 | 143,766,272 | 4.87 |  |
|  | PZE-109106291 | 0.31 | 143,767,731 | 5.36 |  |
|  | PZE-109106296 | 0.43 | 143,769,002 | 5.31 |  |
|  | PZE-109106297 | 0.43 | 143,769,038 | 5.31 |  |
|  | SYN3569 | 0.11 | 143,799,793 | 4.76 |  |
|  | SYN6494 | 0.16 | 143,934,525 | 5.36 |  |
|  | PZE-109106589 | 0.46 | 143,941,650 | 5.26 |  |
|  | PZE-109106743 | 0.42 | 144,168,691 | 4.83 |  |
| 10.01 | SYN4502 | 0.4 | 4,191,188 | 7.25 | None |
|  | SYN4496 | 0.35 | 4,191,766 | 7.24 |  |
|  | PZE-110005408 | 0.23 | 4,191,947 | 7.06 |  |
| 10.03 | PZE-110033464 | 0.46 | 62,841,879 | 5.26 | None |
|  | SYN28404 | 0.46 | 62,855,516 | 5.26 |  |
| 10.03 | SYN26295 | 0.47 | 63,024,092 | 5.26 | None |
| 10.03 | SYN26300 | 0.48 | 63,025,966 | 5.26 | 21210408;195601378;195611413;195599430;194704007;195629925 |
|  | PZE-110033590 | 0.5 | 63,196,862 | 5.22 |  |
|  | PZE-110033612 | 0.32 | 63,288,390 | 5.27 |  |
|  | PZE-110033620 | 0.48 | 63,293,673 | 5.26 |  |
|  | PZE-110033636 | 0.49 | 63,358,042 | 5.26 |  |
|  | PZE-110033642 | 0.29 | 63,364,582 | 5.26 |  |
|  | PZE-110033687 | 0.48 | 63,402,321 | 5.26 |  |
|  | PZE-110033690 | 0.38 | 63,402,722 | 5.25 |  |
|  | PZE-110033702 | 0.39 | 63,404,029 | 5.25 |  |
| 10.03 | PZE-110033833 | 0.29 | 63,586,732 | 5.25 | 195601021;195593940;224030392;195603152;195655882;238008603; |
|  | PZE-110033839 | 0.49 | 63,597,166 | 5.26 | 195613339 |
|  | PZE-110033856 | 0.31 | 63,679,423 | 5.29 |  |
|  | PZE-110033900 | 0.14 | 64,046,250 | 5.26 |  |
|  | SYN29451 | 0.26 | 64,294,620 | 5.27 |  |
|  | PZE-110034047 | 0.42 | 64,297,112 | 5.15 |  |
|  | PZE-110034086 | 0.2 | 64,407,238 | 5.27 |  |
| 10.03 | PZE-110034177 | 0.2 | 64,693,802 | 5.26 | None |
|  | SYN19511 | 0.47 | 64,774,695 | 5.26 |  |
|  | PZE-110034247 | 0.44 | 64,863,008 | 5.27 |  |
| 10.03 | PZE-110034282 | 0.47 | 64,999,008 | 5.26 | 223942852;195622615 |
|  | PZE-110034316 | 0.29 | 65,105,941 | 5.28 |  |
| 10.03 | PZE-110034501 | 0.34 | 65,386,635 | 5.31 | 195628389;195593417;195604969;195652648 |
| 10.03 | PZE-110034862 | 0.23 | 66,073,706 | 5.27 | 195608223 |
|  | PZE-110034877 | 0.23 | 66,077,163 | 5.27 |  |
| 10.03 | PZE-110035024 | 0.28 | 66,366,780 | 5.14 | 223947956;195623347 |
| 10.03 | PZE-110035195 | 0.3 | 66,503,927 | 5.28 | 219885122;195598892 |
| 10.03 | PZE-110035939 | 0.1 | 68,191,798 | 5.27 | 195638947;195610939;195618503 |
|  | PZE-110035940 | 0.2 | 68,191,828 | 5.29 |  |
|  | PZE-110035960 | 0.1 | 68,285,256 | 5.26 |  |
|  | PZE-110036001 | 0.25 | 68,419,825 | 5.03 |  |
|  | PZE-110036027 | 0.14 | 68,497,670 | 5.36 |  |
| 10.03 | PZE-110036140 | 0.38 | 68,757,061 | 5.29 | 195650630 |
| 10.03 | SYN15914 | 0.2 | 68,852,773 | 5.26 | None |
| 10.05 | PZE-110078281 | 0.21 | 132,947,179 | 5.26 | 223973806;238014161;194704495;195606277 |
