# Supplementary material for: Genome-Wide Association Study Identifies Candidate Genes That Affect Plant Height in Chinese Elite Maize (Zea mays L.) Inbred Lines
Source: PLoS One. 2011 Dec 28;6(12):e29229. doi: 10.1371/journal.pone.0029229 (PMC3247246; doi:10.1371/journal.pone.0029229)
Supplement: Table S2 — SNP distribution of the Illumina maizeSNP50 within 277 inbred lines. (DOC) [file pone.0029229.s006.doc]

**Table S2.** SNP distribution of the Illumina maizeSNP50 within 277 inbred lines.

| Chr. | SNP Number | Minor allelic frequency and rate | | | | | | | | | | |
| --- | --- | --- | --- | --- | --- | --- | --- | --- | --- | --- | --- | --- |
|  |  | ≥0.05 | FR(%) | |  | ≥0.1 | FR(%) | |  | ≥0.2 | FR(%) | |
| 1 | 6,905 | 6,432 | 93.15 | |  | 5,931 | 85.89 | |  | 4,564 | 66.09 | |
| 2 | 4,649 | 4,280 | 92.07 | |  | 3,806 | 81.87 | |  | 2,847 | 61.26 | |
| 3 | 5,100 | 4,814 | 94.39 | |  | 4,429 | 86.84 | |  | 3,401 | 66.69 | |
| 4 | 5,003 | 4,591 | 91.76 | |  | 4,110 | 82.15 | |  | 3,134 | 62.64 | |
| 5 | 4,964 | 4,611 | 92.89 | |  | 4,217 | 84.95 | |  | 3,215 | 64.77 | |
| 6 | 3,564 | 3,357 | 94.19 | |  | 3,075 | 86.28 | |  | 2,377 | 66.69 | |
| 7 | 3,709 | 3,422 | 92.26 | |  | 3,171 | 85.49 | |  | 2,404 | 64.82 | |
| 8 | 3,868 | 3,611 | 93.36 | |  | 3,321 | 85.86 | |  | 2,531 | 65.43 | |
| 9 | 3,292 | 3,007 | 91.34 | |  | 2,732 | 82.99 | |  | 2,162 | 65.67 | |
| 10 | 3,181 | 2,976 | 93.56 | |  | 2,737 | 86.04 | |  | 2,099 | 65.99 | |
| Total | 44,235 | 41,101 | 92.92 | |  | 37,529 | 84.84 | |  | 28,734 | 64.96 | |
| Coverage/SNP(Kb) | 46.21 | 49.74 | |  |  | 54.47 | |  |  | 71.14 | |  |
| Gap<100Kb (%) | 84.11 | 83.00 | |  |  | 81.49 | |  |  | 76.79 | |  |

Note: FR, Frequency rate
